# Proteomic and lipidomic analysis of exosomes derived from ovarian cancer cells and ovarian surface epithelial cells

**DOI:** 10.1186/s13048-020-0609-y

**Published:** 2020-01-22

**Authors:** Lin Cheng, Kun Zhang, Yunan Qing, Dong Li, Manhua Cui, Peng Jin, Tianmin Xu

**Affiliations:** 1 The Second Hospital of Jilin University, 218 Ziqiang Street, Changchun, 130000 China; 2 Department of Human Genetics, Emory University School of Medicine, 615 Michael Street, Atlanta, GA 30322 USA

**Keywords:** Ovarian cancer, Exosome, Proteomics, Lipidomics

## Abstract

**Background:**

The limitation of current biomarker of early stage ovarian cancer and the anatomical location of ovarian (depths of the pelvic) make ovarian cancer difficult to be detected in early stage. Growing evidence shows exosomes as key information transmitters, it carried molecules, such as miRNAs, proteins, lipids, double-stranded DNA have been reported as promising biomarkers in many diseases. However, little is known about the protein and lipid composition of ovarian cancer.

**Methods:**

Here, we report proteomic and lipidomic analysis of exosomes derived from ovarian cancer cells (SKOV-3) and ovarian surface epithelial cells (HOSEPiC).

**Results:**

A total of 1433 proteins and 1227 lipid species were identified from two cell line derived exosomes. Several lipid species and proteins significantly differ in SKOV-3 derived exosomes compared to those from HOSEPiC. For example, we noted that ChE and ZyE species were in general more abundant in exosomes from SKOV-3 than from HOSEPiC; Collagen type V alpha 2 chain (COL5A2) and lipoprotein lipase (LPL) were significantly higher in SKOV-3 derived exosomes than HOSEpic (*p* < 0.05).

**Conclusions:**

Our research indicates the promising role of exosomal proteins and lipids in the early diagnosis of ovarian cancer.

## Background

Exosomes are small (30–150 nm diameter) double-membrane bound vesicles that contain several molecules that are specific to the parent cells [1–3]. The mechanism of exosome biogenesis and cargo selection are still unclear, but there are several studies provided precise clues [4–8]. All cells release exosomes either constitutively or upon activation/stress, and tumor cells are released in larger quantity in compared to normal cells [9, 10]. As an information transmitter, exosomes exchange information with distant cells via carrying complex packets stuffed with a selection of proteins, lipids, and nucleic acids. Moreover, exosomes have been shown to play a role in immune response, antigen presentation, cell migration, cell differentiation, tumor invasion and other aspects [11]. During the development of cancer, exosomes released from cancer cells are able to transfer a variety of molecules, including those that are cancer-specific, to other cells so as to manipulate their environment, making it more favorable for tumor growth and invasion [11, 12]. Recent studies implicate that exosomes can mediate drug resistance in various intracellular processes [13].

Since exosomes have been explored from a variety of bodily fluids, including urine [14], saliva [15], blood [9] and cerebrospinal fluid [16], milk [17], and the double-membrane structure provide a shelter to multiple bioactive molecule which avoids degradation. Thus, exosomes seem as a vehicle that is full of ideal non-invasive biomarkers with great potential in the detection of oncogenesis, tumor spread, and drug resistance. However, data attributing to cancer specific intercellular transfer molecules to exosomes are still limited. The structure and function of membranes and domains are determined by the assembled molecular lipids and membrane-bound proteins. In-depth characterization of exosomes will help to elucidate their precise biological functions.

Ovarian cancer (OC) is the most fatal gynecologic malignancy worldwide [18, 19]. Because of the lack of early diagnostic markers [20], almost 50% of OC is diagnosed in women over the age of 65 [21], the majority of ovarian cancer patients are diagnosed in an advanced stage [22]. Hence, to address this question, we performed a comparative analysis of the protein and lipid composition of 2 different cell line derived exosomes. We chose an ovarian cancer (SKOV-3) and an ovarian surface epithelial (HOSEPiC) cell line because 70% ovarian cancer origin of ovarian germinal epithelium. Our results reveal enriched proteins and pathways, potentially involved in intercellular communication, and an extraordinary sorting of lipids into exosomes which may dependent on its original.

## Materials and methods

### Cell culture

An human ovarian surface epithelial cell line (HOSEPiC) and an ovarian cancer cell SKOV-3 were cultured in 37 °C in 5% carbon dioxide and RMPI-1640 medium supplemented with 10 and 15%, EV-depleted fetal bovine serum (FBS was centrifuged overnight at 120,000 g to pellet out vesicles), 100 units/ml penicillin and 100 μg/ml streptomycin.

### Exosome isolation

Cells were incubated with EV-depleted medium for 2 days to reach approximately 80% confluency and supernatants were collected for exosomes isolation using classical differential ultracentrifugation methods [23] with tiny modification. Briefly, the supernatant was centrifuged to remove dead cells, cell debris and microvesicles at 300 g for 10 min, 2000 g for 10 min and 10,000 g for 30 min. The supernatant was concentrated using a 100 KDa molecular weight cut-off centrifugal filter (Millipore, Germany). The concentrated suspension was centrifuged in a SW41 ultracentrifuge rotor at 110,000 g for 80 min. In order to avoid missing exosomes, approximately 2 ml of supernatant was left. These volumes were mixed and exosomes were pelleted in a SW41 rotor at 110,000 g for 80 min. The supernatant was gently removed and the exosome pellet was washed with 11 mL PBS solution. Exosomes was pelleted again by a third round of ultracentrifugation was with the same parameters. Exosomes used for proteomic analysis were resuspended in 30 ul of SDT buffer (4% SDS, 100 mM DTT, 150 mM Tris-HCl pH 8.0). Exosomes used for flow cytometry analysis (FACS), TEM and size analysis were resuspended in PBS. Exosomes used for lipidomic were resuspended in 200 ul of pure water.

### Transmission electron microscopy

Pelleted exosomes were resuspended in PBS. 5ul resuspended exosomes were loaded onto 150 mesh copper grids and stood at RT for 5 min. Excess liquid was removed using filter paper. The exosome containing grids were air-dried and 5ul 2% phosphotungstic acid was used to stained exosomes at RT for 5 min. Excess liquid was removed using filter paper again and the stained exosome containing grids were air-dried and observed under the electron microscope at 80 kV.

### Flow cytometry analysis

As previously described with tiny modification, exosomes were attached to 4um aldehyde/sulphate latex beads (Invitrogen) by mixing 30 μg exosomes in a 10 ul volume of beads in a 1.5 ml microcentrifuge for 15 min at room temperature. This suspension was diluted to 1 ml with PBS and incubated on a tube rotator overnight at 4 °C. The free binding sites were saturated by adding 110ul of 1 M glycine and left stood on the branch at room temperature for 30 min. Exosomes-bound beads were washed three times in 0.5% BSA in PBS and centrifuged for 4 min at 4000 g. The bead pellet was responded in 0.5 ml 0.5% BSA in PBS. 10ul coated bead were incubated with 5ul anti-Human CD63 and CD9 (12–0639, eBioscience, 11–0098-42, Invitrogen) antibody diluted with 45ul 0.5% BSA in PBS 30 min at 4 °C. For each measurement a total number of 10,000 events were recorded.

### Size analysis of exosomes

Pelleted exosomes were resuspended in PBS and analyzed using NANO ZS 90 (NanoSight, Malvern, UK) according to the manufacture.

### Western blot

All cells were harvested upon completion of 2 days in EV-depleted medium. The cell pellets were washed twice with ice-cold PBS and lysed immediately with the lysis buffer (Protein Extraction Mammalian Total Protein Extraction Kit, Transgen Biotech) maintained at 4 °C for 30 min. Cellular debris was removed by centrifugation (14,000 g, 10 min at 4 °C). Exosome suspensions were used for protein quantification directly, and protein amount was determined using a BCA protein assay kit (Beyotime Biotechnology). Denaturing SDS-Polyacrylamide gel electrophoresis was performed in 12% acrylamide gels using equivalent total protein. Separated proteins were transferred onto polyvinylidene difluoride membranes through electroblotting. Western blots were performed using primary and secondary antibodies coupled to HRP, diluted according to the suppliers’ recommendations and detected using an enhanced chemiluminescence (ECL) system. The antibodies used were anti-FASN (ABclonal, A0462), anti-L1CAM (ABclonal, A8555), anti-TSG101 (Proteintech, 14,497–1-AP), anti-GAPDH (Proteintech, 60,004–1-Ig), and anti-β-Actin (Proteintech, 60,008–1-Ig). All antibodies were raised in rabbit, exceptβ-Actin and GAPDH that were raised in mouse.

### Proteomics

3 replicate samples of exosomes from each source cell type were used for proteomic analysis. The detergent, DTT and other low-molecular-weight components in protein samples were removed using UA buffer (8 M Urea, 150 mM Tris-HCl pH 8.0) by repeated ultrafiltration (Microcon units, 10 kD). Then 100 μl iodoacetamide (100 mM IAA in UA buffer) was added to block reduced cysteine residues and the samples were incubated for 30 min in darkness. The filters were washed with 100 μl UA buffer three times and 100 μl 25 mM NH4HCO3 buffer twice. Finally, the protein suspensions were digested with 4 μg trypsin (Promega) in 40 μl 25 mM NH4HCO3 buffer overnight at 37 °C, and the resulting peptides were collected as a filtrate. The peptides of each sample were desalted on C18 Cartridges (Empore™ SPE Cartridges, Sigma), concentrated by vacuum centrifugation and reconstituted in 40 μl of 0.1% (v/v) formic acid. The peptide content was estimated by UV light spectral density at 280 nm using an extinctions coefficient of 1.1 of 0.1% (g/l) solution that was calculated on the basis of the frequency of tryptophan and tyrosine in vertebrate proteins. The peptide mixture was loaded onto a reverse phase trap column (Thermo Scientific Acclaim PepMap100, 100 μm*2 cm, nanoViper C18) connected to the C18-reversed phase analytical column (Thermo Scientific Easy Column, 10 cm long, 75 μm inner diameter, 3 μm resin) in buffer A (0.1% Formic acid) and separated with a linear gradient of buffer B (84% acetonitrile and 0.1% Formic acid) at a flow rate of 300 nl/min controlled by IntelliFlow technology. MS analysis was performed on a Q-Exactive mass spectrometer (Thermo Scientific) that was coupled to Easy nLC (Thermo Scientific) for 120 min.

### Lipidomics

6 replicate samples of exosomes from each source cell type were used for lipidomic analysis. Exosome pellets were frozen at − 80 °C and transferred to APT (Shanghai) on dry ice for lipid composition analysis. Samples thawed at 4 °C environment slowly, 200 ul pure water and 240ul ice-cold methanol were added and vortex mixed. 800 ul MTBE were added and vortex mixed. The mixed solution was placed for 20 min at room temperature. Then the mixed solution was centrifuged at 8000 g for 15 min at 10 °C. The upper organic phase was taken, and nitrogen was blown dry. The dried powder was re-resuspended in 200ul isopropyl before UPLC-MS. The samples were separated by UHPLC Nexera lc-30a system (Column temperature 45 °C, Flow rate at 300 uL/min, sample size 2ul). Mobile phase composition A: 10 mM ammonium formate acetonitrile aqueous solution (acetonitrile: Water =6:4, v/v), B: 10 mM ammonium formate acetonitrile isopropanol solution (acetonitrile: isopropanol =1:9, v/v). MS analysis was performed on a Q-Exactive mass spectrometer (Thermo Scientific). The mass spectrometer was operated in positive ion mode. MS data was acquired using a data-dependent top10 method dynamically choosing the most abundant precursor ions from the survey scan (300–1800 m/z) for HCD fragmentation.

### Analysis

The proteomic MS data were analyzed using MaxQuant software version1.5.3.17 (Max Planck Institute of Biochemistry in Martinsried, Germany) [24] against the UniProt complete human proteome protein sequence database (version: 2018-01-15, number of sequences: 161,584.). Searches were performed with fragment ion mass tolerance of 20 ppm, maximum missed cleavage of 2 and carbamidomethylation of cysteine was specified as a fixed modification and oxidation of methionine as variable modification. Peptide False discovery (≦0.01) was set. Only protein groups identified with at least two or more peptides (sum of razor and unique) were carried forward in the analysis. Label-free quantification of proteins was performed via the LFQ method in Maxquant software. All statistical analyses were performed using R version 3.4. We also used gene ontology (GO) and KEGG pathway enrichment analyses to annotate biological function to proteins enriched in exosomes.

The lipidomic MS data were quantified using an LipidSearch software(Thermo Scientific™)for producing lipid identification and peak alignment (precursor tolerance: 5 ppm, product tolerance: 5 ppm, product ion threshold: 5%). Data showed RSD > 30% were deleted. We selected the data which missing value were below 50% in the group to analyze using an SIMCA-P 14.1 software (Umetrics, Umea, Sweden). Quality control samples were used to monitor the overall quality of the lipid extraction and MS analyses. We also visualized the normalized protein and lipid profiles of exosomes on heatmaps.

## Results

### Characterization of exosomes derived from two ovarian cell lines

**Fig. 1:**
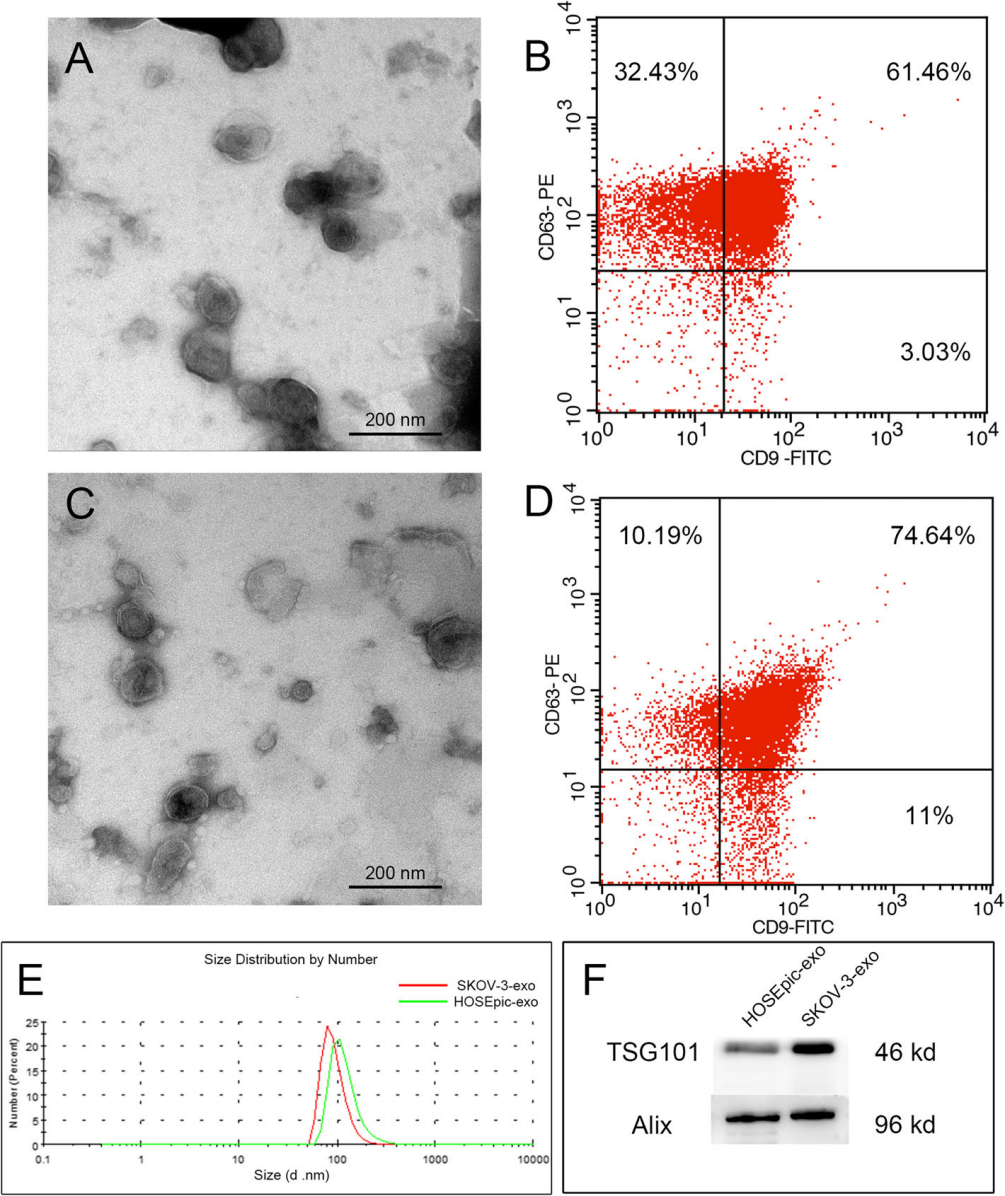
Exosome isolation and analysis. A&C Representative transmission electron microscopy (TEM) images obtained for exosomes from HOSEPiC and SKOV-3. Scale bar: 100 nm. B&D flow cytometry analysis analyses show intensities for exosomal markers (CD9, CD63). (E) Particle size distribution by NanoSight analysis of exosomes from HOSEPiC and SKOV-3 (F) Western blot analyses show increased intensities for exosomal markers (Alix, TSG101)

The exosomes secreted by SKOV-3 and HOSEPiC cells were isolated from EV-depleted medium by a combination of differential centrifugation. Exosomes obtained were extensively characterized using several methods such as western blot and flow cytometry analysis to identify specific exosomal markers, electron microscopy and NanoSight to identify specific exosomal structure and size (Fig. 1).

### In-depth proteomics of exosomes derived from two ovarian cell lines

**Fig. 2:**
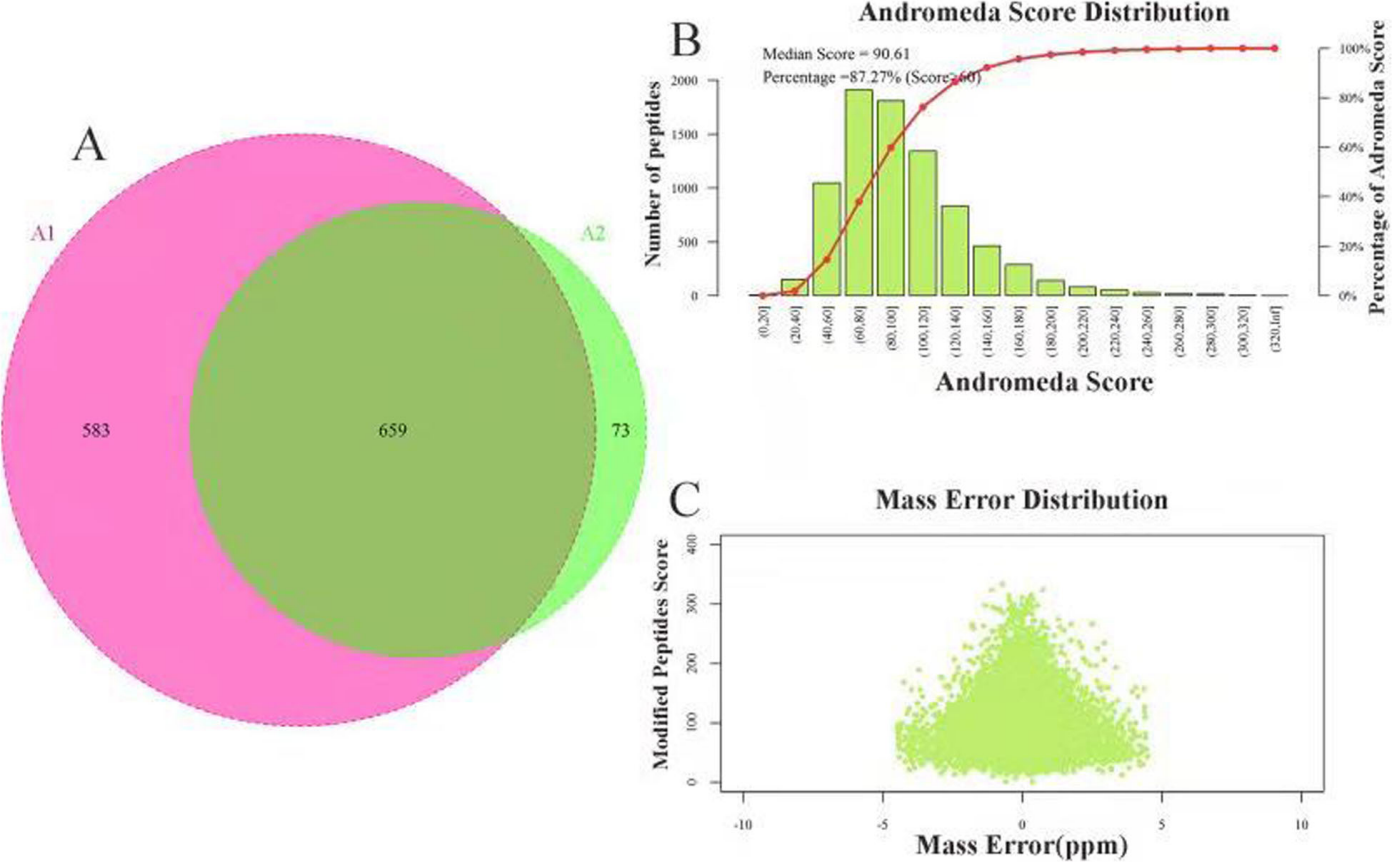
**a** Comparison of exosome proteome from HOSEPiC and SKOV-3. **b** Andromeda score distribution **c** Distribution of mass deviation of peptide ions

**Fig. 3:**
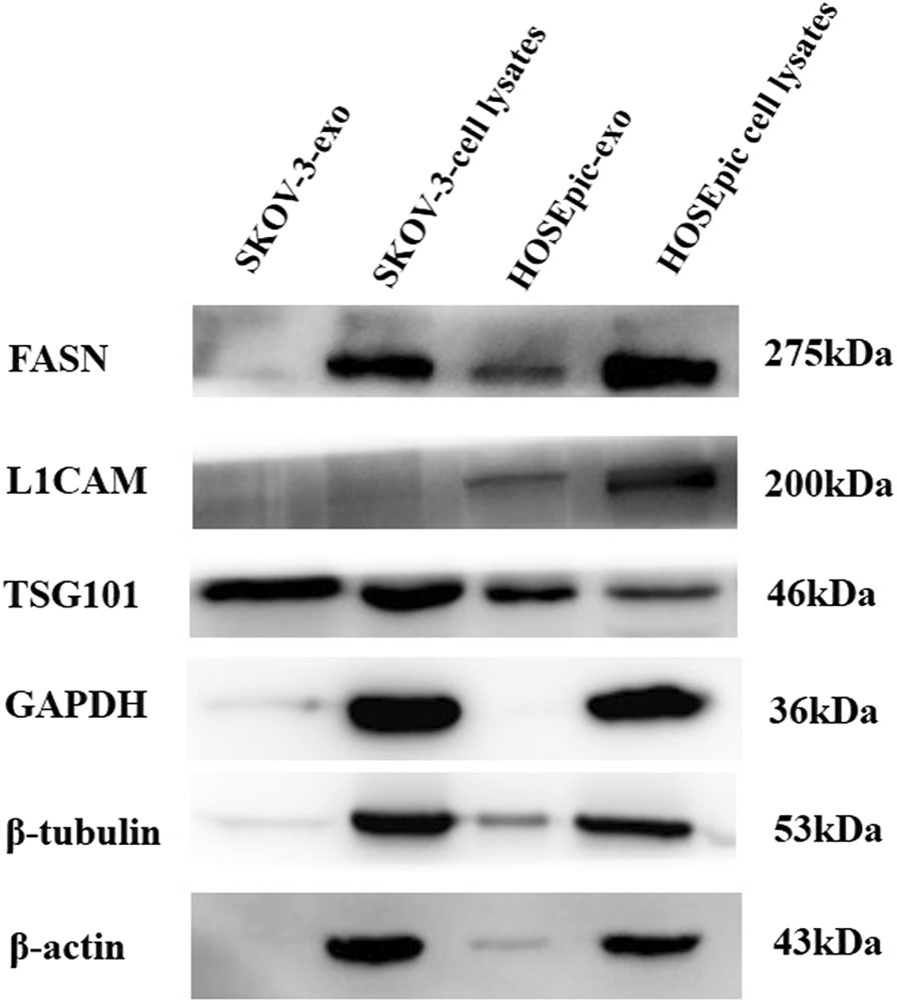
Validation of the MS/MS results by Western blotting. Western blot analyses show increased intensities for exosomal marker (TSG101) and lower intensities for negative control markers (β-Actin, β-tubulin and GAPDH) in exosomes. FASN and L1CAM was only verified in exosomes derived from HOSEPiC as identified by MS/MS

Using rigorous peptide and protein identification criteria total of 1433 proteins groups were identified from exosomes derived from SKOV-3 and HOSEPiC cells (732 proteins in SKOV-3 derived exosomes and 1242 proteins in HOSEPiC derived exosomes, Additional file 1: Table S1). 659 proteins were identified in the exosomes from both cell lines (Fig. 2a). Key to this project was the use of the nano-flow UPLC coupled to a Q-Exactive mass spectrometer. The quality deviations of all identified peptide segments were mainly distributed within 10 ppm, indicating that the identification results were accurate and reliable. We used Andromeda to analyze and grade MS spectrum showed in Fig. 2. Western blot signal intensities for exosome markers TSG101 were greater in the exosome fraction compared to the cell lysates. FASN and L1CAM was only verified in exosomes derived from HOSEPiC as identified by MS/MS. GAPDH, β-Actin andβ-tubulin were not verified in exosomes by Western-blot although it was identified by MS/MS in both cell lines (Fig. 3).

### Differentially expressed proteins and function analysis

**Fig. 4:**
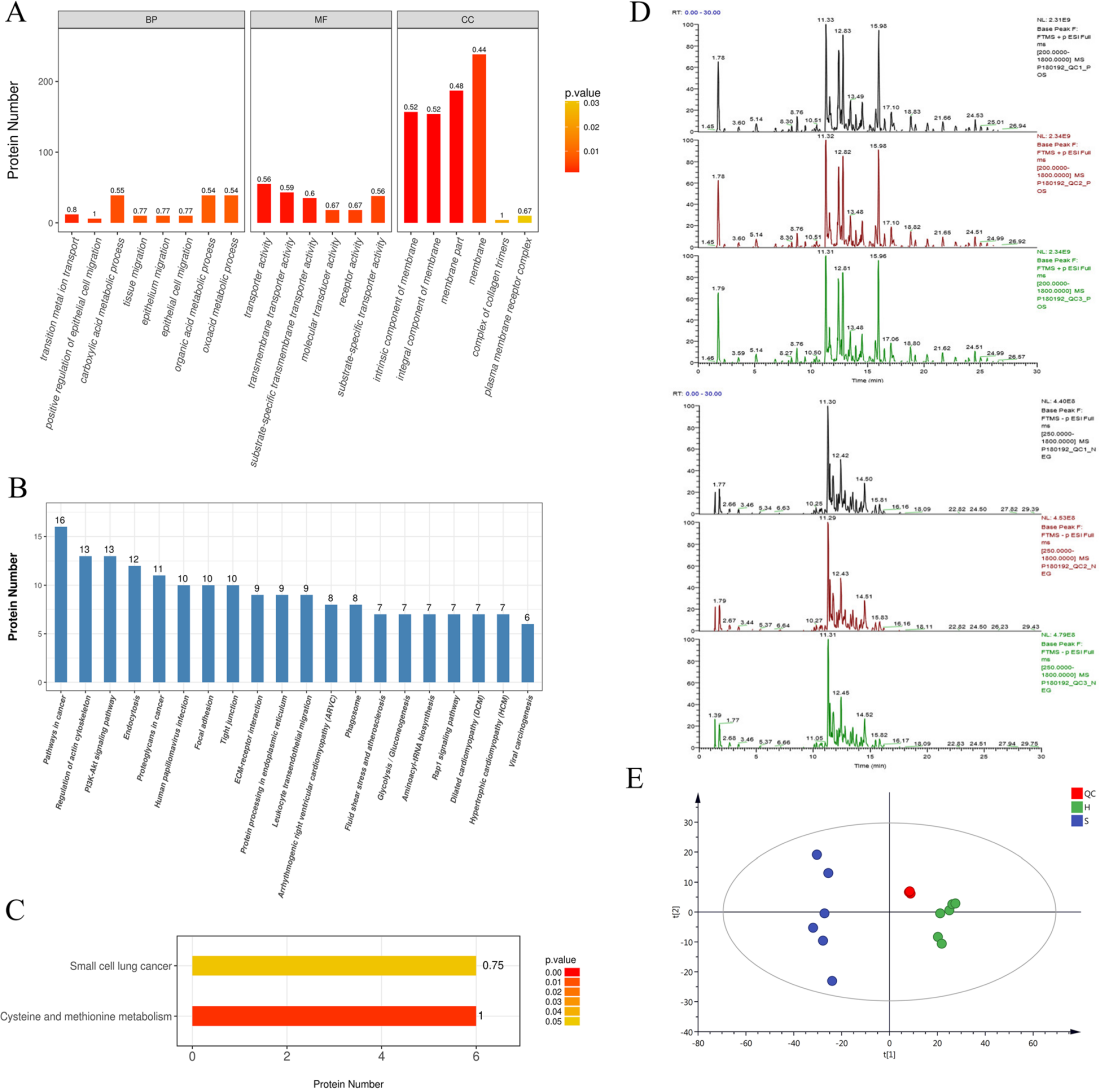
**a** Gene ontology (GO) analysis on differentially expressed proteins (Top 20). Bar chart color represent *P* value based on Fisher’s Exact Test, the closer the color is to red, the smaller the P value is. BP, biological process; MF, molecular function; CC, cellular component. **b** KEGG analysis differentially expressed proteins (Top 20). **c** KEGG analysis of differentially expressed proteins shows significantly differences in cysteine and methionine metabolism, and small cell lung cancer related pathway. **d** UHPLC-Obitrap MS BPC for three runs of quality control samples shows high precision. **e** PCA analysis shows a good experimental repeatability

To further analyze this observation, we sub-screening differentially expressed proteins. Furthermore, only those proteins identified in 2 or more biological replicates in both cell line derived exosomes, with a *P* value of less than 0.05, and which fold change greater than 2.0 times (up more than 2 times or down less than 0.5 times were included in the profile differentially expressed in abundance. Those proteins identified in 2 or more biological replicates in only one cell line derived exosomes were included in consistent presence / absence expression profile (Fig. 4, Additional file 2: Table S2). To be pointed out, collagen type V alpha 2 chain (COL5A2) and lipoprotein lipase (LPL) were significant higher in SKOV-3 derived exosomes than HOSEpic (*p* < 0.05). COL5A2 has been reported as specific predictive signature for the diagnosis and prognosis of pancreatic cancer [25] and bladder cancer [26].We also visualized the differentially expressed proteins on heatmaps and used gene ontology (GO) analysis (Blast2Go, https://www.blast2go.com/) to annotate biological function. Statistics showed the significant variation exosomal proteins derived from the two different cell lines involved in the following biological processes and molecular functions: transition metal ion transport, positive regulation of epithelial cell migration, carboxylic acid metabolic process, epithelial cell migration, transporter activity, transmembrane transporter activity, molecular transducer activity and receptor activity (Fig. 4). The FASTA protein sequences of differentially expressed proteins were blasted against the online Kyoto Encyclopedia of Genes and Genomes (KEGG) database (http://geneontology.org/) to retrieve their KOs and were subsequently mapped to pathways in KEGG [27]. The corresponding KEGG pathways were extracted and showed in Fig. 4. In brief, pathways of cysteine and methionine metabolism and small cell lung cancer have shown significant differentially expressed.

### Lipidomic analysis showed high reproducibility

To assess the reproducibility of our experiments, the samples of each group were mixed into quality control samples (QC) in equal quantity. The analysis results of the samples of QC UHPLC-obitrap MS base peak were compared for overlapping spectra, as shown in the Fig. 4, which shows that the response intensity and retention time of each chromatographic peak overlapped substantially, indicating that the experiment is relatively repetitive. PCA analysis was performed on all experimental samples and QC samples after pareto-scaling. As shown in Fig. 4, QC samples are closely clustered and located in the middle of each group, indicating that the lipidomic analysis showed high reproducibility.

### Characterization of lipid composition

In order to identify lipid species differentially expressed in exosomes derived from SKOV-3 and HOSEPiC cells, 6 replicate samples of exosomes from each source cell type were analyzed. In total, 30 lipid classes, 1227 lipid species were identified (Additional file 3: Table S3). In particular, SKOV-3 derived exosomes contained higher levels of GM3, ZyE, LPI, LPC, AcCa, LPS, LPG and ChE, lower levels of Cer, DGDG, PS, PI, PG, SM, PE, DG and CerG3 than exosomes derived from HOSEPiC cells (*p* < 0.05), whereas more similar levels of other lipid classes were found (Additional file 4: Table S4). To be mentioned, no significant differences were found in TG, of which decreased levels were found to be a specific metabolic feature foreshadowing an early relapse in epithelial ovarian cancer (EOC) patients plasma lipidomics study [28].

### Important lipid alterations of two ovarian cell lines derived exosomes

**Fig. 5:**
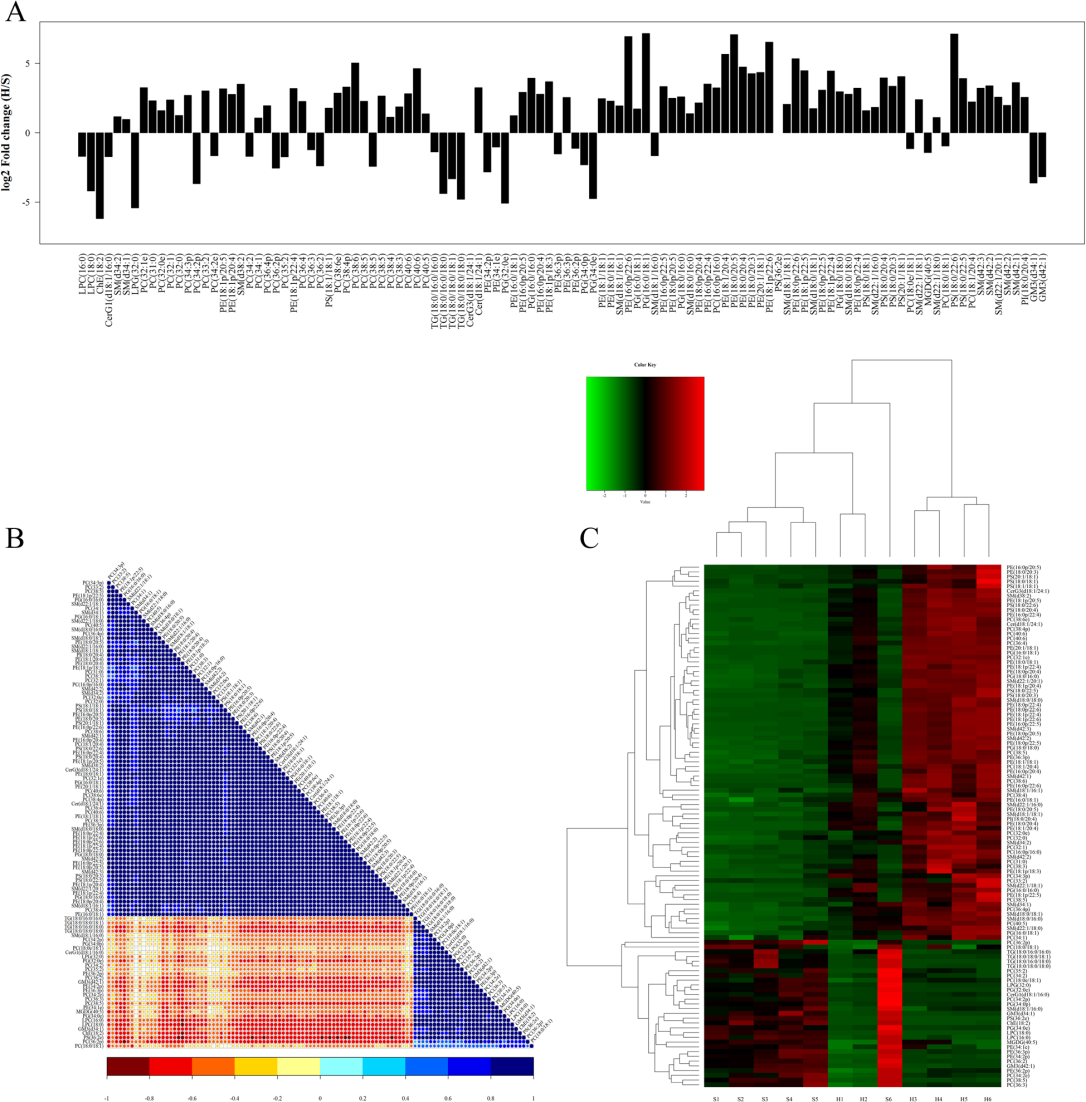
**a** Analysis of lipid variation multiple with significant difference. =**b** Lipid correlation analysis of significant difference. **c** Cluster Analysis of lipidomic results

To further analyze lipidomic data, variable weight for the projection (VIP) was used to measure the impact strength and interpretation ability of each lipid expression pattern on the classification and discrimination of each group, and univariate statistical analysis was further performed to verify the significance of the difference in lipids. In this experiment, VIP > 1 and *P* value < 0.05 was used as the screening standard, and the significant differences between each group was screened out and listed in Additional file 3: Table S3. In total, 110 lipid species were screened out as potential lipid biomarkers of EOC, which were presented in Fig. 5a and also listed in Table 1. To be interesting, some lipid species were reported by an early relapse biomarker in EOC [28], such as LPC(18:0), PC(36:3), PC(38:6), PC(40:5), PC(38:6). We also used correlation analysis to help measure the degree of correlation between lipid molecules (Fig. 5c).

**Table 1:** Identified differential lipid species between two ovarian cell lines derived exosomes

LipidIon	CalMz	RT (min)	Fold Change	*P*-value	OPLS-DA VIP	QCRSD
LPC(16:0) + H	496.33977	3.3190213	3.2401429	0.0003273	1.1373	0.0297514
LPC(18:0) + H	524.37107	4.7209243	18.269889	0.0003722	2.60482	0.0162352
ChE(18:2) + NH4	666.61836	22.834863	72.791038	0.0003399	2.89397	0.011065
CerG1(d18:1/16:0) + H	700.5722	11.699674	3.2940782	0.0306673	1.14636	0.0045913
SM(d34:2) + H	701.5592	10.295418	0.4433831	0.0076993	1.16119	0.0658362
SM(d34:1) + H	703.57485	11.59398	0.5114373	0.0367638	1.5197	0.0020378
LPG(32:0) + H	709.5378	12.101539	42.668799	0.0141787	2.26234	0.0588612
PC(32:1e) + H	718.57452	12.887364	0.1043478	0.0001296	2.05444	0.0049736
PC(31:0) + H	720.55378	11.846352	0.2007092	0.0013034	1.24693	0.0262676
PC(32:0e) + H	720.59017	13.029801	0.3277517	0.0079895	1.90627	0.0053236
PC(32:1) + H	732.55378	11.612277	0.1923281	0.0046353	2.73062	0.0462711
PC(32:0) + H	734.56943	12.360385	0.4178622	0.0068592	3.52812	0.0127862
PC(34:3p) + H	740.55887	11.488	0.1531682	0.0021939	1.15239	0.0675083
PC(34:2p) + H	742.57452	12.158077	12.690591	0.0175789	2.15496	0.0040868
PC(33:2) + H	744.55378	12.829097	0.1222581	0.0301204	1.49374	0.0676107
PC(34:2e) + H	744.59017	12.28901	3.1371582	0.0077942	1.67519	0.0831756
PE(18:1p/20:5) + H	748.52757	11.625209	0.1097354	0.0004896	1.32269	0.0171607
PE(18:1p/20:4) + H	750.54322	12.501618	0.145539	0.000242	1.19719	0.0083309
SM(d38:2) + H	757.6218	12.532221	0.087633	0.000192	1.0073	0.0213935
PC(34:2) + H	758.56943	11.630869	3.2335929	0.0364593	5.7643	0.1027676
PC(34:1) + H	760.58508	12.445472	0.4729024	0.0141368	5.97923	0.0810498
PC(36:4p) + H	766.57452	11.940753	0.2565588	0.0008954	1.12283	0.0060599
PC(36:2p) + H	770.60582	12.349255	5.8753359	0.0001111	1.20156	0.1285883
PC(35:2) + H	772.58508	12.248583	3.3185929	0.0435064	1.71303	0.0021791
PE(18:1p/22:4) + H	778.57452	13.294718	0.1085165	4.293E-05	1.13316	0.0007308
PC(36:4) + H	782.56943	11.487673	0.2070776	0.0001643	2.85354	0.0116555
PC(36:3) + H	784.58508	11.748421	2.3365561	0.0049429	3.18958	0.0241481
PC(36:2) + H	786.60073	12.677741	5.2284223	0.0029346	7.57054	0.0179213
PS(18:1/18:1) + H	788.54361	11.642431	0.2881103	0.0102609	1.25586	0.0394853
PC(38:6e) + H	792.59017	11.988893	0.1371336	0.0005692	1.11168	0.0054911
PC(38:4p) + H	794.60582	12.934363	0.1005617	7.065E-05	1.04556	0.0036017
PC(38:6) + H	806.56943	11.187251	0.0307172	0.000384	1.03144	0.0063008
PC(38:5) + H	808.58508	11.810332	0.2048139	2.768E-05	1.17442	0.2107591
PC(38:5) + H	808.58508	12.672307	5.3756449	0.0003799	2.10979	0.0183503
PC(38:5) + H	808.58508	11.552074	0.1578107	0.0010544	2.61177	0.0658505
PC(38:4) + H	810.60073	12.488833	0.4547764	0.0017399	2.12696	0.001785
PC(38:3) + H	812.61638	12.668413	0.272076	0.0008442	1.10512	0.028863
PC(40:6) + H	834.60073	11.597658	0.141325	0.0001152	1.03595	0.0098316
PC(40:6) + H	834.60073	12.20053	0.0405773	5.149E-05	1.72933	0.0220091
PC(40:5) + H	836.61638	12.501089	0.3850075	0.0014969	1.6471	0.0468449
TG(18:0/16:0/16:0) + NH4	852.80147	23.939109	2.5892204	0.0391508	1.59551	0.0019084
TG(18:0/16:0/18:0) + NH4	880.83277	24.519245	20.899098	0.0029053	3.16115	0.0048875
TG(18:0/18:0/18:1) + NH4	906.84842	24.538863	10.011565	0.0111865	1.50329	0.012747
TG(18:0/18:0/18:0) + NH4	908.86407	25.00585	27.739567	0.0034531	2.08332	0.046467
CerG3(d18:1/24:1) + H	1134.7874	14.091305	0	0.0001224	1.07113	0.0066018
Cer(d18:1/24:1) + HCOO	692.61985	16.175473	0.103872	0.0001471	1.25519	0.0215203
PE(34:2p)-H	698.51302	12.449142	7.0769491	0.0006763	3.93889	0.0324108
PE(34:1e)-H	702.54432	13.438345	2.0385687	0.0113353	1.156	0.0088682
PG(32:0e)-H	707.52325	12.091773	33.793704	0.0061178	6.58985	0.0156174
PE(16:0/18:1)-H	716.52358	12.720163	0.4202182	0.0004424	1.83386	0.0108465
PE(16:0p/20:5)-H	720.49737	11.583383	0.1314003	0.0051701	1.44299	0.0107055
PG(16:0/16:0)-H	721.50251	11.473539	0.0657456	1.681E-05	7.95788	0.0536726
PE(16:0p/20:4)-H	722.51302	12.248596	0.1447987	2.026E-05	4.13162	0.073243
PE(18:1p/18:3)-H	722.51302	11.479	0.0774059	0.0007414	2.93232	0.0263587
PE(36:3p)-H	724.52867	12.689641	2.8785532	0.0012986	1.82325	0.0338385
PE(36:3p)-H	724.52867	12.527277	0.1696422	1.244E-05	1.10348	0.0758919
PE(36:2p)-H	726.54432	13.506758	2.1755145	0.0056018	3.72929	0.0298701
PG(34:0p)-H	733.5389	12.13369	4.956339	0.0246676	2.58981	0.0648588
PG(34:0e)-H	735.55455	13.058572	26.79393	0.0073044	1.85088	0.0811271
PE(18:1/18:1)-H	742.53923	12.800264	0.1802797	2.189E-05	4.40304	0.107758
PE(18:0/18:1)-H	744.55488	13.783132	0.2032096	0.0005934	2.90082	0.0055799
SM(d18:1/16:1) + HCOO	745.55013	10.268659	0.2587852	0.003933	1.11216	0.0391053
PE(16:0p/22:6)-H	746.51302	11.933663	0.0081673	0.0009601	2.06975	0.037648
PG(16:0/18:1)-H	747.51816	11.589231	0.2997752	0.0061899	1.95396	0.0031406
PG(16:0/18:1)-H	747.51816	12.082	0.0070412	0.0001049	1.70948	0.0101288
SM(d18:1/16:0) + HCOO	747.56578	11.143139	3.1337131	0.0163085	1.20354	0.1452831
PE(16:0p/22:5)-H	748.52867	12.289308	0.0987534	8.502E-05	3.64684	0.0068211
PE(18:0p/20:5)-H	748.52867	12.567227	0.1766982	0.0002361	1.83825	0.009736
PG(18:0/16:0)-H	749.53381	12.425961	0.1646077	5.421E-05	2.56412	0.0199873
SM(d18:0/16:0) + HCOO	749.58143	11.7146	0.3821158	0.0016634	1.98624	0.0581853
PE(18:0p/20:4)-H	750.54432	13.279505	0.2216461	5.222E-05	3.07046	0.0127945
PE(16:0p/22:4)-H	750.54432	12.97263	0.0866529	3.018E-05	1.93611	0.0087864
PC(16:0p/16:0) + HCOO	762.56545	12.873873	0.1047239	0.0025272	1.16224	0.0074952
PE(18:1/20:4)-H	764.52358	11.833072	0.0198255	2.089E-05	2.0106	0.0174142
PE(18:0/20:5)-H	764.52358	12.138	0.0074497	0.0003435	1.20561	0.0241654
PE(18:0/20:4)-H	766.53923	12.75693	0.0375343	4.79E-06	3.30905	0.1177161
PE(18:0/20:3)-H	768.55488	13.193352	0.0522966	0.003736	1.16198	0.0061066
PE(20:1/18:1)-H	770.57053	13.784833	0.0493772	0.0002845	1.56243	0.0107648
PE(18:1p/22:6)-H	772.52867	12.002327	0.010826	0.0002546	1.51339	0.0270652
PS(36:2e)-H	772.5498	11.450658	#DIV/0!	7.631E-05	1.33306	0.0013688
SM(d18:1/18:1) + HCOO	773.58143	11.463026	0.2381133	0.0003138	1.47317	0.0137748
PE(18:0p/22:6)-H	774.54432	12.944895	0.0248033	7.927E-05	1.9671	0.0044874
PE(18:1p/22:5)-H	774.54432	12.319588	0.0451147	0.0005202	1.26561	0.0308905
SM(d18:0/18:1) + HCOO	775.59708	12.38504	0.2961012	0.0003765	2.84705	0.0099288
PE(18:0p/22:5)-H	776.55997	13.285239	0.1177648	2.278E-05	1.95952	0.0080996
PE(18:1p/22:4)-H	776.55997	13.012059	0.0459673	6.588E-05	1.21688	0.0024014
PG(18:0/18:0)-H	777.56511	13.412865	0.1266703	4.927E-05	1.43803	0.0305797
SM(d18:0/18:0) + HCOO	777.61273	12.769534	0.1442123	4.697E-05	1.23292	0.0171988
PE(18:0p/22:4)-H	778.57562	14.047477	0.107071	2.975E-05	1.1974	0.0061423
PS(18:0/18:1)-H	788.54471	12.473056	0.3286841	0.0297073	2.66766	0.016332
SM(d22:1/16:0) + HCOO	803.62838	13.458944	0.2784275	0.0002187	1.11001	0.0696966
PS(18:0/20:4)-H	810.52906	11.58011	0.0645448	0.0001105	1.45732	0.0093627
PS(18:0/20:3)-H	812.54471	11.97813	0.0967219	0.0004739	1.80055	0.0917789
PS(20:1/18:1)-H	814.56036	12.487242	0.0604681	0.002872	1.35476	0.0169701
PC(18:0e/18:1) + HCOO	818.62805	14.214201	2.2124072	0.0456916	1.2273	0.0203368
SM(d22:1/18:1) + HCOO	829.64403	13.588027	0.1901319	0.002038	1.56036	0.0645059
MGDG(40:5)-H	831.59917	12.658	2.6826723	0.0060226	3.97242	0.0061761
SM(d22:1/18:0) + HCOO	831.65968	14.596852	0.4635037	0.007412	1.82634	0.0147283
PC(18:0/18:1) + HCOO	832.60731	12.864	1.924755	0.0336785	1.82967	0.0768031
PS(18:0/22:6)-H	834.52906	11.311759	0.0071894	0.0001123	1.4428	0.00347
PS(18:0/22:5)-H	836.54471	11.595335	0.0667759	0.0001863	1.29217	0.0258396
PC(18:1/20:4) + HCOO	852.57601	11.52204	0.2125022	5.243E-05	1.16355	0.0111386
SM(d42:3) + HCOO	855.65968	13.525602	0.1063234	0.0001879	2.9138	0.0199491
SM(d42:2) + HCOO	857.67533	13.545112	0.0942641	9.483E-05	1.18221	0.0006172
SM(d22:1/20:1) + HCOO	857.67533	14.526616	0.167393	0.000104	5.06469	0.0136233
SM(d42:2) + HCOO	857.67533	14.727306	0.252808	0.001211	1.75892	0.023403
SM(d42:1) + HCOO	859.69098	14.966437	0.0806861	0.0003376	1.01072	0.0182043
PI(18:0/20:4)-H	885.54986	11.369021	0.1675608	0.0003305	2.32953	0.0672784
GM3(d34:1)-H	1151.7059	9.9060133	12.292786	0.001588	1.16125	0.126017
GM3(d42:1)-H	1263.8311	13.772293	8.9902052	0.0008371	1.08214	0.0259905

## Discussion

Exosomal molecules, such as miRNA, protein, lipid, double-stranded DNA have been reported as promising biomarkers in pancreatic cancer [29], prostate cancer [14], pheochromocytoma [30], Stroke [31] and other diseases. Initially studies by Thomas [32] and Shen [33] had reported exosomal proteome profiles derived from different ovarian cancer cells in starvation conditions. In this article, we provide the in-depth proteomic and lipidomics analysis of exosomes derived from ovarian cancer cells and ovarian surface epithelial cells with EV-depleted medium and focus more on the metabolic perspective. As the venn diagram showed, high overlapped were found between two exosomal proteins. To obtain a systematic insight into the proteome profiles, we analyzed the significant differentially expressed proteins by GO and KEGG. The most enriched pathway were cysteine and methionine metabolism pathway. The content of various amino acid metabolic enzymes differentially expressed including L-lactate dehydrogenase, adenosylhomocysteinase, branched-chain-amino-acid aminotransferase, aspartate aminotransferase, and malate dehydrogenase. A proteomic profiling of plasma exosomes also found serine-type endopeptidase activity changed significantly in EOC patients [34], in the meantime, they found 10 genes (among the 50 differentially expressed genes) participated in the complement and coagulation cascade. However, we did not find any coagulation-related differentially expressed genes in our study. As the latest study demonstrated that neural stem/progenitor cell (NSC)-derived EVs function as independent metabolic units that are able to modify the concentrations of critical nutrients, with the potential to affect the physiology of their microenvironment [35], suggesting the low level of cysteine and methionine metabolic enzymes in tumor derived exosomes might be in favor of establishment of tumor microenvironment [12]. Lin et al. [36] observed that Glucose-6-phosphate dehydrogenase, transketolase and transaldolase 1, three key enzymes regulated pentose phosphate pathway, were all marked in the same exosomal parts of proteins between two late-stage ovarian cell lines, OVCA429 and HO8910PM. However, our data did not show these three key enzymes enrichment in SKOV-3 derived exosomes. But we observed that about 30% of differentially expressed proteins between 2 cell lines were participated in metabolic process. Interestingly, we also found lipoprotein lipase, a crucial node in the management of plasma lipid levels by promoting hydrolytic cleavage of the triglyceride core of lipoproteins, was rise significantly in SKOV-3 derived exosomes, this might be the reason for the plasma lipid species variation in malignant or borderline ovarian tumors, and benign pathology [28, 37].

Exosomes originate from the late endosomal compartment and transport their cargo extracellularly to communicate with other cells. It appears that exosome lipid composition is unique and does not reflect the composition of the plasma membrane [38]. Exosomes are enriched in lipids such as PCs and PEs and bioactive lipids involved in signaling such as SM, Cers, cholesterol, lysophosphatidylcholine, among others [39]. So far, there was no report about the lipidomics analysis of exosomes, either from ovarian cancer or in the context of ovarian surface epithelial cell line. We successfully identified a total of 1227 lipid species exosomal lipids from two ovarian cell lines by performing an LC–MS/MS workflow.

In terms of lipid classes, we noted that ChE and ZyE species were in general more abundant in exosomes from SKOV-3 than from HOSEPiC. It is interesting that resistin, a positive regulation of steroid hormone secretion protein, was only identified in exosomes derived from SKOV-3, since some proteins related with response to steroid hormone and steroid metabolic process were more abundant in exosomes from HOSEPiC. Aberrant regulation of cholesterol homeostasis has been associated with multiple types of cancer [40]. Moreover, numerous studies have shown increased levels of cholesterol in tumors as compared to normal tissue [40, 41], some suggesting cholesterol may accumulate in tumor tissue [42, 43]. As we observed, similarly to tumor tissue, both cholesterol and its precursor zymosterol were both accumulate in tumor derived exosomes. It is not clear why this is the case, it is possible that the exosomal steroid concentration is cell type dependent and/or depends on the transfer information packaged by the exosome-secreting cells [14]. Unfortunately, no similar results were found in EOC patient’s serum or plasma samples [37]. This may be due to the complicated sources of lipid composition in serum or plasma, of which only a minority of lipid metabolic changes due to ovarian cancer, therefore focuses on the exosomes can show the advantages: the tumor cells secrete exosomes exhaustedly, most exosomes in peripheral blood are tumor derived, and can better reflect the lipid metabolic disorder of tumor, thus supporting the early diagnosis of ovarian cancer.

Lysophosphatidic acid has been proposed to be involved in various cancers through different pathogenesis [44]. For example, LPE causes migration and the invasion of ovarian cancer cells [45]; LPS suppress T lymphocyte proliferation [46], and stimulates the migration of colorectal cancer cells and glioma cells [47, 48]. In our study, we found that exosomes from HOSEPiC were more abundant in PS, PI, PE, PG, while exosomes from SKOV-3 were more abundant in LPI, LPS, LPG, LPC, suggesting that the exosomal lipids play an important role in the progress of tumor invasion and metastasis. To our knowledge, Urban et al. [49] have observed the similar results. Their lipidomic studies presented on the urinary exosome lipid repertoire in control and renal cell carcinoma patient, and showed lysophospholipids were the largest differences lipid classes.

In terms of lipid species, we successfully identified 1212 species in exosomes from HOSEPiC and 1202 species in exosomes from SKOV3. More details were list in Additional file 3: Table S3 for the species number relationship. In total, 110 lipid species were screened out with significant differences between each group. The highest significance were PG(34:1)-H and ChE(18:2) + NH4. Furthermore, some lipid species showed species specificity, such as CerG3(d42:2) + H were only identified in exosomes from HOSEPiC, PS(36:2e)-H were only identified in exosomes from SKOV3, indicating that the potential use of exosomal lipid species as cancer biomarkers. 5 lipid species also reported by Li and his group in a lipidomic study of plasma from 70 EOC patients [28].

In conclusion, this study shows that exosomal lipids and protein are promising cancer biomarkers. Several lipid species and proteins significantly differ in SKOV-3 derived exosomes compared to those from HOSEPiC. Further experiments will have to be performed in clinical specimens to validate these results. Thus demonstrating their diagnostic potential, additional specimens will have to be included: For example, exosomes derived from tissues and peripheral blood of benign ovarian tumor patients and borderline ovarian tumor patients, to investigate the specificity and use of these biomarkers in early diagnosis, and patients at different stages of disease to investigate if they can be used to indicate the process of malignant tumor.

## Supplementary information

**Additional file 1: Table S1.** Identified proteins from two ovarian cell lines derived exosomes.

**Additional file 2: Table S2.** consistent presence / absence expression protein profile.

**Additional file 3: Table S3.** Identified lipid species from two ovarian cell lines derived exosomes.

**Additional file 4: Table S4.** Identified lipid classes from two ovarian cell lines derived exosomes.

## Data Availability

The datasets used and/or analyzed during the current study are available from the corresponding author on reasonable request.

## Abbreviation

Cer: Ceramides
CerG: Simple Glc series
ChE: Cholesterol Ester
EV: Extracellular vesicles
GM: Gangliosides
LPC: Lysophosphatidylcholine
LPC: Lysophosphatidylcholine
LPG: Lysophosphatidylglycerol
LPI: Lysophosphatidylinositol
LPS: Lysophosphatidylserine
MGDG: Monogalactosyldiacylglycerol
PC: Phosphatidylcholine
PE: Phosphatidylethanolamine
PG: Phosphatidylglycerol
PI: Phosphatidylinositol
PS: Phosphatidylserine
SM: Sphingomyelin
TG: Triglyceride
ZyE: Zymosterol
